# Relationship between certain serum biochemical values and serostatus against *Anaplasma marginale* in dairy cows

**DOI:** 10.14202/vetworld.2019.1858-1861

**Published:** 2019-11-26

**Authors:** Myassar O. Alekish, Zuhair Bani Ismail

**Affiliations:** Department of Veterinary Clinical Sciences, Faculty of Veterinary Medicine, Jordan University of Science and Technology, Irbid 22110, Jordan

**Keywords:** blood parasites, dairy cows, hemolysis, metabolic diseases

## Abstract

**Aim:**

This study was conducted to evaluate the possible association between values of certain serum biochemical parameters and seropositivity against *Anaplasma marginale* in dairy cows.

**Materials and Methods:**

Serum samples from 60 seropositive and 40 seronegative cows were used to determine the values of beta-hydroxybutyrate (BHB), glucose, creatinine, blood urea nitrogen, total protein, albumin, alkaline phosphatase (ALP), aspartate aminotransferase (AST), alanine transaminase (ALT), lactate dehydrogenase (LDH), and gamma-glutamyl transferase (GGT) using commercially available kits and reagents. The serostatus of cows against *A. marginale* was determined using a commercially available cellular enzyme-linked immunosorbent assay kit according to the manufacturer’s recommendations. Significant differences in serum biochemical values between seropositive and seronegative groups were evaluated using independent Student’s t-test. Possible associations between the serostatus of the cows and different biochemical parameters were evaluated using univariate followed by multivariate logistic regression analyses.

**Results:**

There was a statistically significant increase (p≤0.05) in values of total protein, BHB, LDH, and AST in seropositive cows compared to seronegative cows while a non-significant increase in values of ALP, ALT, and GGT was detected in seropositive cows. A strong correlation (R=0.69) between serum levels of BHB, LDH, and AST and seropositivity against *A. marginale* was detected.

**Conclusion:**

There is evidence of a possible association between *A. marginale* infection and liver damage/hepatic fatty degeneration in dairy cows. Further studies, however, are required to elucidate the exact pathophysiological mechanisms of this relationship.

## Introduction

*Anaplasma marginale*, the causative agent of anaplasmosis, is tick-borne, obligate intraerythrocytic rickettsial bacteria [1-3]. The disease is considered endemic in most tropical and subtropical regions of the world, including South and Central America, the USA, Europe, Africa, Asia, the Middle East, and Australia [2,4]. The disease affects cattle primarily, but buffalo, sheep, goats, and wild ruminants can also be infected [2]. Worldwide, the estimated prevalence of anaplasmosis has been found to vary between 30% in endemic areas and 100% in certain tropical regions [5].

Anaplasmosis is capable of causing significant economic losses due to high morbidity, mortality, reduced production, and losses associated with treatment and control measures [2,6]. In dairy herds, several risk factors have been identified, including season, age, sex, breed, reproductive management, milk yield, stocking density, flies, and *Rhipicephalus microplus* infestation [7,8].

Ticks are considered the primary host and a reservoir for *A. marginale* [4]. Ticks usually become infected after feasting on blood of an infected cow [9]. In ticks, the bacteria replicate primarily in the salivary tissues and, subsequently, transmit the disease while feeding on the blood of a normal cow [10]. In cows, the bacteria infect the erythrocytes and endothelial cells [11]. While young cattle are asymptomatic, clinical signs in adults may include fever, weight loss, abortion, and eventually death within an average of 4 weeks after exposure [4]. Surviving cattle usually develop protective immunity but become long-term carriers of the microorganism [6].

In recent literature, there has been a significant body of research concerning the prevalence, epidemiology, and pathogenesis of anaplasmosis in different parts of the world [12,13]. However, no research can be cited regarding serum biochemical alterations in *Anaplasma* seropositive cattle. Therefore, the objectives of this study were to evaluate the possible associations between *A. marginale* serostatus of dairy cows and certain serum metabolic values including beta-hydroxybutyrate (BHB), glucose, creatinine, blood urea nitrogen, total protein, albumin, alkaline phosphatase (ALP), aspartate aminotransferase (AST), alanine transaminase (ALT), lactate dehydrogenase (LDH), and gamma-glutamyl transferase (GGT) in adult dairy cows.

## Materials and Methods

### Ethical approval and informed consent

All required institutional ethical approvals were obtained before this study was conducted. Written and signed consents were obtained from farm owners before blood samples were collected.

### Animals

A total of 100 cows were used in this study. Cows belonged to small to medium (50-500 cows) dairy herds. Cows were randomly selected from the chosen herds. Before sample collection, cows were subjected to a complete physical examination. Only apparently healthy cows were included in the study. All cows were kept in open sheds on dirt with adequate shaded areas provided and fed total mixed ration. Freshwater was freely available at all times. Cows were routinely milked 2 or 3 times/day.

### Sample collection

Approximately 10 ml of whole blood was aseptically collected from the coccygeal vein using hypodermic needles and plain tubes. Samples were transported in an icebox to the laboratory within 3-4 h after collection. Serum was harvested by centrifugation of blood samples at 5000× g for 10 min and stored at −20°C until further analysis was carried out.

### Laboratory analysis

The serostatus of the cows involved in the study against *A. marginale* was determined using a commercially available cellular enzyme-linked immunosorbent assay kit according to the manufacturer’s instructions (VMRD; USA). The microplates were read at 620 nm wavelength using spectrophotometer (Thermo Fisher Scientific; USA). The inhibition percentage (% I) was calculated according to the equation: % I = 100 [1-(Sample Optical Density ÷ Negative Control Optical density)]. According to the kit instructions, positive samples have inhibition of ≥30% while negative samples have <30% inhibition.

For the serum biochemical analysis, the following parameters were determined using commercially available kits and reagents according to the manufacturer’s instructions: BHB (BioVision; USA), glucose, creatinine, blood urea nitrogen, total protein, albumin, ALP, AST, ALT, LDH, and GGT (Cayman Chemical; USA). Data were compared to previously published normal values in normal lactating dairy cows [14,15].

### Statistical analysis

For statistical analysis, cows were divided into two groups according to the serostatus against *A. marginale*: Seropositive and seronegative groups. The associations between the serostatus and different biochemical parameters were initially screened using Pearson’s Chi-square test followed by univariate and multivariable logistic regression analyses. Independent Student’s t-test was used to evaluate significant differences in the evaluated blood parameters between seropositive and seronegative cows. p≤0.05 was considered statistically significant. Statistical analysis was performed using SPSS software version 23 (IBM Corp., N.Y., USA).

## Results

All cows involved in the study were apparently healthy at the time of sample collection. The serum concentrations of glucose, blood urea nitrogen, creatinine, total protein, and albumin in seropositive and seronegative cows against *A. marginale* are presented in Table-1 [14,15]. There was a statistically significant increase (p≤0.05) in values of total protein in seropositive cows compared to seronegative cows. There were no other significant changes in any of the other parameters.

**Table-1:** Serum concentrations of glucose, blood urea nitrogen, creatinine, total protein, and albumin in seropositive and seronegative cows against *Anaplasma marginale*.

Parameters	Serostatus	Mean±SD	Reference values [14,15]	Percentage within normal range
Glucose (mg/dl)	+	59±17	31-77	97
	−	66±22		95
Blood urea nitrogen (mg/dl)	+	16±1.5	10-25	97
	−	15±3		92
Creatinine (mg/dl)	+	0.5±2	0.4-1.0	99
	−	0.9±3		98
Total protein (g/l)	+	88±10*	60-80	36
	−	60±15		95
Albumin (g/l)	+	29±5	30-36	99
	−	32±5		99

* p≤0.05

**Figure-1:**
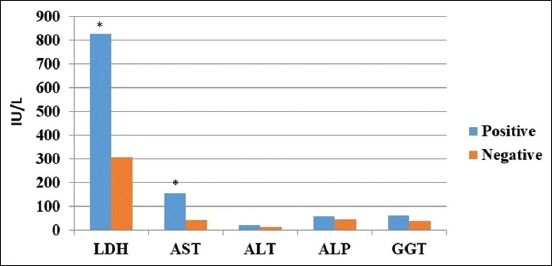
The serum activities of alkaline phosphatase, aspartate aminotransferase, alanine transaminase, lactate dehydrogenase, and gamma-glutamyl transpeptidase in seropositive and seronegative cows against *Anaplasma marginale* (*indicates p≤0.05).

The serum activities of ALP, AST, ALT, LDH, and GGT in seropositive and seronegative cows against *A. marginale* are presented in Figure-1. A statistically significant (p≤0.05) increase in values of LDH and AST was detected in seropositive cows compared to seronegative cows. There was a non-significant increase in values of ALP, ALT, and GGT in seropositive cows compared to seronegative ones. The mean±SD values of LDH and AST in seropositive cows were 827±138 IU/L and 155±60 IU/L, respectively, while in seronegative cows, these values were 306±110 and 42±4 IU/L, respectively. Values of ALP, ALT, and GGT in seropositive cows were 59±10 IU/L, 20±3 IU/L, and 60±6 IU/L, respectively, while in seronegative cows, these values were 45±5 IU/L, 12±2, and 40±3 IU/L, respectively.

**Figure-2:**
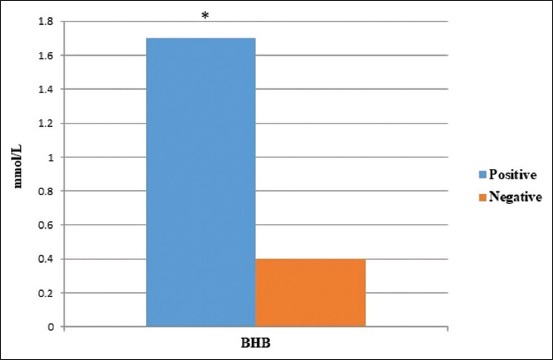
Serum levels of beta-hydroxybutyrate in seropositive and seronegative dairy cows against *Anaplasma marginale* (*indicates p≤0.05).

Serum levels of BHB in seropositive and seronegative dairy cows against *A. marginale* are presented in Figure-2. There was a statistically significant (p≤0.05) increase in serum values of BHB in seropositive cows compared to seronegative cows. A strong correlation (R=0.69) was detected between serum levels of BHB, LDH, and AST and seropositivity against *A. marginale*.

## Discussion

Dairy cows become under significant metabolic stress around parturition, leading to remarkable immunosuppression and increased susceptibility to various diseases [16,17]. Studying the association between diseases and their effects on the general health status, welfare, and productivity of the cow is a vital tool to understand their pathophysiology and therefore developing effective control strategies. In this study, a strong association between certain serum metabolic parameters and the serostatus against *A. marginale* in dairy cows was detected and reported for the 1^st^ time. It has been suggested that dairy cows are most susceptible to clinical anaplasmosis in the immediate postpartum period due to the state of immunosuppression characteristic of this period [17]. This was fortified in this study by the finding of a significant increase in serum levels of BHB and a strong correlation between seropositivity and BHB levels. BHB is considered a marker of negative energy balance in dairy cows just after parturition which is associated with substantial immunosuppression [18-20].

In the serum biochemical analysis, total protein concentrations were found significantly elevated in seropositive cows while all other parameters were within normal limits. This increase in total protein could be due to increased globulin concentrations in chronically infected cows [21].

Serum liver enzyme activities are indicative of liver function and integrity [22]. In this study, all liver enzymes including AST, LDH, ALP, ALT, and GGT were elevated, but values were only significantly increased for AST and LDH. These findings are similar to those previously published in dairy cows naturally infected with *A. marginale* [23,24]. In cattle, increased AST usually accompanied by a significant increase in serum levels of creatinine kinase is associated with muscular damage due to increased laying times and recumbency in acutely affected cows with anaplasmosis [22]. However, in chronically affected cows, this rise in AST and LDH levels is most likely due to hypoxic hepatic cellular damage. In addition, the increased serum levels of AST and ALT could also be due to myocardial or erythrocytic damage [24].

## Conclusion

The results of this study provide evidence of the association between *A. marginale* infection and possible liver damage/hepatic fatty degeneration in lactating dairy cows. Further studies, however, are warranted to elucidate the exact pathophysiological mechanism of this relationship.

## Authors’ Contributions

MOA research concept and design. ZBI data interpretation and manuscript writing. Sample collection and laboratory analysis. All authors read and approved the final manuscript.
